# Graph isomorphism-based algorithm for cross-checking chemical and crystallographic descriptions

**DOI:** 10.1186/s13321-023-00692-1

**Published:** 2023-02-23

**Authors:** Andrius Merkys, Antanas Vaitkus, Algirdas Grybauskas, Aleksandras Konovalovas, Miguel Quirós, Saulius Gražulis

**Affiliations:** 1 Sector of Crystallography and Chemical Informatics, Institute of Biotechnology, Life Sciences Center, Vilnius University, Vilnius, Lithuania; 2 Department of Biochemistry and Molecular Biology, Institute of Biosciences, Life Sciences Center, Vilnius University, Vilnius, Lithuania; 3 Departamento de Química Inorgánica, Universidad de Granada, 18071 Granada, Spain

**Keywords:** Molecular graphs, Graph isomorphism, SMILES, Crystallography Open Database

## Abstract

Published reports of chemical compounds often contain multiple machine-readable descriptions which may supplement each other in order to yield coherent and complete chemical representations. This publication presents a method to cross-check such descriptions using a canonical representation and isomorphism of molecular graphs. If immediate agreement between compound descriptions is not found, the algorithm derives the minimal set of simplifications required for both descriptions to arrive to a matching form (if any). The proposed algorithm is used to cross-check chemical descriptions from the Crystallography Open Database to identify coherently described entries as well as those requiring further curation.

**Supplementary Information:**

The online version contains supplementary material available at 10.1186/s13321-023-00692-1.

## Introduction

Reliable knowledge about the structure and properties of chemical compounds is essential for many branches of science. With the advent of computer-based analyses and the increase of computational power, open collections of machine-readable descriptions of chemical compounds, such as ChEMBL [1] and the Crystallography Open Database (COD [2]), become more and more important.

Chemical and crystallographic information is generally collected using different experimental methods and recorded in separate datasets using different data formats. Due to this, the correspondence between the two datasets is not always outright evident. Chemical attributes such as atomic connectivity, bond orders, atomic charges or the presence of lone electron pairs are either known beforehand at the experiment design stage or are derived at the material identification stage. Some of the most popular machine-readable data formats used to record this type of chemical information include the Structure Data File (SDF), the Chemical Markup Language (CML [3]) as well as several linear representations like SMILES [4], InChI [5] and chemical names in IUPAC notation [6, 7]. Similarly, crystallographic information such as crystal lattice parameters and 3D atomic coordinates are determined during crystallographic experiments and are usually recorded using the machine-readable Crystallographic Information File (CIF [8, 9]) data format. Although SDF, CML and CIF formats could in principle record both the chemical and crystallographic properties simultaneously, they rarely do so in supplementary material of peer-reviewed publications.

Because of this separation of chemical and crystallographic knowledge, sometimes the complete picture can only be achieved by aligning data from files in different formats. However, both manual and automated alignment is hindered by the lack of mapping between molecules and atoms in these files. Moreover, even the overlapping information is occasionally contradictory. Detection and correction (if possible) of such contradictions improves the quality of data.

In this publication we propose a method, based on molecular graph isomorphism, for overlaying different chemical representations of the same crystal, collected from various crystallographic and chemical data sources. The proposed method identifies matching representations as well as reveals differences between them. We also present here a compendium of COD entries that provides insight into both the coherently described entries and entries having differences in their representations. In “Methods” we provide the details of our approach and in “Results and discussion” we evaluate it by aligning machine-readable representations from crystal structure publications as deposited in the COD and ChEMBL.

## Methods

### Data sources

**Fig. 1:**
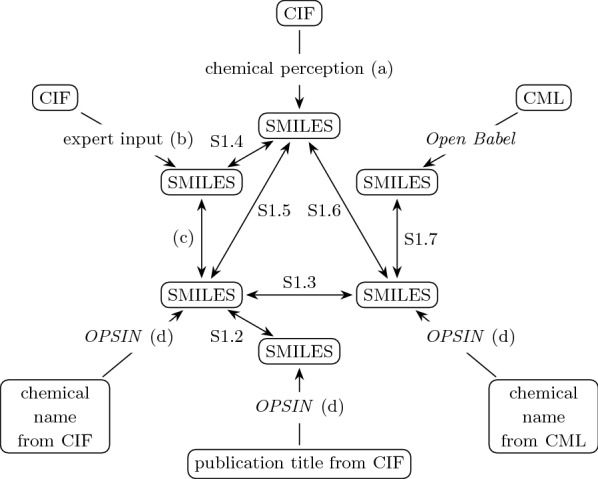
Data flow diagram representing origins of the data, their conversions and performed comparisons. Rounded rectangles represent pieces of data and single direction arrows represent their transformations, with transforming processes and tools named on the arrows. Bidirectional arrows represent performed comparisons between SMILES with references to sections describing them. Letters in parentheses are used as shorthand references to the relevant sections and peer-reviewed publications: (a) “Chemical perception of crystallographic data”, (b) [11], (c) “Comparison of curated and chemical name-derived SMILES”, (d) “Interpretation of chemical names”. Prefix S denotes sections of Additional file 1

In this study, we have analysed crystal structure descriptions from the COD, the largest open-access crystallographic database. Being a crystallographic database, the COD primarily consists of crystal descriptions in CIF format. To make these descriptions suitable for chemical analysis, chemical perception has to be performed to infer chemical attributes from the atomic coordinates. The workflow we have used for this task is described in more detail in “Chemical perception of crystallographic data”. Furthermore, additional chemical representations can be extracted from CIF files or found in the supplementary material of related peer-reviewed publications. CIF files rarely describe chemical structures, but they may contain IUPAC chemical names, either as values of the _chemical_name_systematic data item or included in the publication titles. These chemical names can be processed by *OPSIN*, a software tool capable of converting IUPAC chemical names into SMILES and CML representations [10] with a reasonably high level of reliability [11]. Processing of chemical names is described in more detail in “Interpretation of chemical names”. In addition to the chemical names, we used the manually curated collection of SMILES representations [11] which covers nearly half of the entries in the COD. Supplementary materials in CML format were also consulted as they usually contain chemical annotations not available in CIF files. All used data sources and their conversions into comparable representations are shown in Fig. 1.

It must be noted that publications quite often lack explicit mapping between the main paper text and supplementary data files (e.g. by using matching identifiers or mapping tables). In publication texts, chemical compounds are typically numbered sequentially or a number-letter combination is used to identify compounds (such as ‘2a’, ‘2b’, etc.). However, in supplementary data files, such as CIF or CML, these identifiers are no longer used. CIF data block names or CML <molecule> element id attributes have different values, assigned to them by crystallographers or chemists without coordination with the main publication text. Such lack of explicit mapping makes it rather difficult to automatically match chemical entities described in the main text and in the supplementary data files, especially when dealing with large datasets.

For the above mentioned reason, we do not attempt to establish the mapping between supplementary CIF and CML files based on their identifiers. Instead, we assume that CIF and CML files originating from the same publication describe the same structure or set of structures (see Section S1.3 of Additional file 1).

### Definitions

Crystal structures in scientific literature are usually reported in CIF format, which is also used by the COD. Each crystal structure contains one or more *molecular entities* [12], which for the purpose of this study we define as groups of atoms held together by covalent bonds or equivalent forces. A molecular entity may consist of a single atom and may also be charged. In this study each crystal structure is represented by a set of distinct molecular entities that excludes:

- crystallographic translation equivalents;
- symmetry equivalents acquired by applying chirality preserving crystal symmetry operations;
- chemically identical molecular entities: tautomers, cis/trans isomers and enantiomers are perceived as different, but rotamers are not.

Thus, when comparing the contents deduced from two different representations of a crystal, we are essentially comparing two sets of distinct molecular entities.

**Fig. 2:**
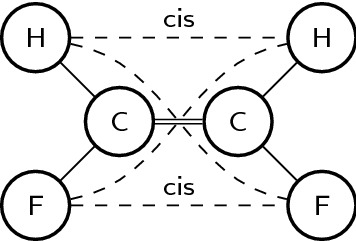
Representation of the cis/trans configuration of a double bond. Dashed edges are added to identify cis and trans relations between attached atoms. Cis edges are labelled “cis”, remaining two dashed lines are trans (labels not shown)

**Fig. 3:**
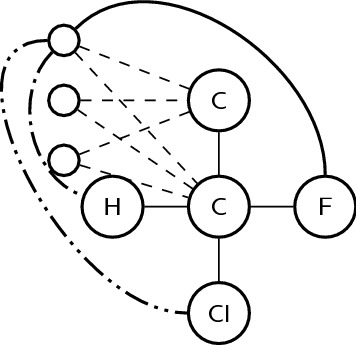
Representation of a tetrahedral chiral centre (partial graph shown). Small empty circles are named *enumeration-listing nodes*, representing three possible alternative enumerations of the remaining three attached atoms in clockwise order. Dashed edges connect enumeration-listing nodes to the central atom and the atom from which the central atom is viewed. Dashed and dotted edges connect enumerator-listing nodes with enumerated atoms, with number of dots on edge identifying the order (H atom is first, Cl is second and F is third). The remaining six dashed and dotted edges are not shown for clarity of the figure. In addition, the other nine enumerator-listing nodes and related edges are not shown

To apply graph-based algorithms on molecular entities, we have to define how molecular entities are represented by graphs. We have based our representation on a widely used approach of representing each molecular entity as a connected graph with atoms corresponding to vertices and bonds to edges [13]. Chemical attributes such as atom chemical type and charge are represented as vertex attributes. Similarly, bond order is represented as an edge attribute. We have used additional vertices and edges to represent cis/trans configurations of double bonds and chirality settings, drawing inspiration from Faulon’s [14] methods for encoding atom and bond attributes in simple graphs. In our approach, the cis/trans configuration of a double bond is represented by joining all atoms attached to opposite sides of the bond with edges having attributes “cis” or “trans” (four edges in total, see Fig. 2 for a schematic example). Chirality setting is represented by providing all possible clockwise enumerations of the remaining three attachments when looking from each of the four chiral centre attachments (see Fig. 3 for a schematic example). This way the same chiral centre is encoded by the same subgraph regardless of the atom order in the input.

### Isomorphism

In cheminformatics, graph isomorphism between molecular entities is usually established by comparing their canonical representations (invariants exhausting all chemical attributes). It is known that graphs with identical canonical representations are isomorphic [14, 15]. At the heart of a canonical representation lies the canonical labelling problem. Having it solved, string representations can be written using SMILES or other similar techniques. InChI and InChIKey representations have been developed for canonical representation specifically and are used to collate molecular entities in databases such as ChEMBL [16]. Nevertheless, successful applications of canonical labelling in SMILES have also been reported [17].

Both the isomorphism and canonical labelling in graphs depend on functions that are used to compare graph vertices and edges. Generally, a set of vertex (atom) and edge (bond) attributes are taken into consideration, disregarding other attributes. The original Morgan algorithm [18] uses only the degree sums of adjacent graph vertices to create vertex invariants. This method does not take into consideration many important atom characteristics, such as chemical types, and extending the algorithm to include these details requires substantial modifications. *CANGEN* canonicalisation algorithm, as presented by Weininger et al. [19], ignores bond orders and requires additional passes for cyclic molecular entities. The InChI algorithm (at least in versions 1.04 and earlier) does not involve the inspection of atom charges, isotopes and bond orders when establishing the canonical order of atoms. Sometimes this results in canonicalisation failures [17].

In our study, we use various chemical attributes to distinguish vertices and edges. Chemical element, charge and isotope attributes are considered when comparing vertices. Bond order attribute is considered when comparing edges. Stereochemistry, i.e. chirality of tetrahedral centres and cis/trans configuration of double bonds, is encoded using special vertices and edges, as described in “Definitions”.

Solutions for molecular graph isomorphism and canonical labelling problems are known at least since Faulon (1998) [14]. Faulon’s algorithm treats all vertices and edges as equal, thus additional overhead is required to encode atom and bond attributes using special vertices and edges. We were unable to find a free and open source implementation of this algorithm and therefore employed the *nauty* [15] library to perform canonical numbering of graph vertices. An implementation of *nauty*’s algorithm is already used in InChI [20]. As *nauty* does not support edge attributes (it treats all edges as equal), we simulate the difference by introducing special vertices to carry bond attributes. To conveniently interface *nauty* with the *cod-tools* software package and the rest of our infrastructure which is predominantly written in Perl, we have developed a Perl binding package called *Graph::Nauty* [21]. The developed tools allow access to both the canonical labelling and the isomorphism functionalities, nevertheless we only used the canonical labelling for this study. We have additionally checked all the graphs whose canonical representations match to make sure they are isomorphic.

### Molecular entity comparison

When comparing two sets of distinct molecular entities, correspondence between these entities in the sets has to be established. An intuitive method of doing so is comparing every molecular entity from one set to every molecular entity from the other set. Such method has the complexity of $O(N^2)$, where *N* is the number of molecular entities in the larger of the two sets, thus it becomes inefficient for large *N*. A more efficient method is to assign a string representation “key” (e.g. SMILES or InChI) for each molecular entity and look for matching keys in the compared sets. In this work we used SMILES representations as the keys, however, certain conventions had to be introduced to turn them into canonical graph representations. Rules for doing so are described in “Canonical representation in SMILES”.

In the simplest case, when the two molecular entity sets are outright equal, the algorithm simply matches the SMILES keys and reports the corresponding molecular entities as identical. Otherwise, molecular entities undergo simplifications (modifications of underlying molecular graphs) until either their representations become identical or the list of simplifications is exhausted. In the former case, the minimal list of changes producing identical representations (isomorphic molecular graphs) is found, showing that the differences between the sets of molecular entities can be accounted for, or explained, automatically. In the latter case, sets of molecular entities are considered different. The following simplifications are performed by our comparison software:

1. Removal of chiral markers ($s_{@}$)—chiral markers can sometimes be omitted as information about the chirality may be absent or deemed unimportant by researchers or software.
2. Removal of cis/trans markers ($s_{ct}$)—same as with chiral markers, cis/trans markers may also be absent for the same reasons.
3. Removal of charge ($s_\pm$)—differences in charge assignment may occur due to missing hydrogen atoms or ions, researchers may choose to omit charges of zwitterions, etc.
4. Conversion of all bond orders to single covalent ($s_b$)—bond orders can be misplaced due to inconsistencies and variability in molecular geometry.
5. Removal of aromaticity setting from atoms ($s_a$)—discrepancies in aromaticity assignment may occur due to variability in molecular geometry or due to the alternative resonance forms.
6. Removal of hydrogen atoms ($s_h$)—hydrogen atom attachments may vary due to the lack of information about bond types or misplaced charges.
7. Removal of atom types ($s_e$)—atom types sometimes are misassigned, thus overall connectivity itself is interesting to study.

All combinations of these simplifications ($2^7$ in total) are tested in a fixed order to establish the smallest set of the least drastic changes required to arrive to isomorphic representations. In our software, each combination of simplifications is encoded as a sequence of 7 binary digits by the *bin*() function. Each binary digit in the representation generated by *bin*() corresponds to the presence (1) or absence (0) of a simplification in the same order as provided in the list above, with $s_{@}$ corresponding to the least singnificant bit and $s_e$ to the most significant bit. For example, 0001001 would stand for the removal of charges and chiral markers. All the combinations are split into three categories that come in the following order:

1. Combinations that preserve both the atom types and the hydrogen atoms (simplifications 1–5).
2. Combinations that remove the hydrogen atoms, but preserve the types of the remaining atoms. Removing hydrogen atoms may affect chiral and cis/trans markers, thus this simplification is deferred until after others have been tried.
3. Combinations that disregard the atom types. Atom types are ignored only after all other combinations are exhausted as the last resort attempt to detect molecular entities with possibly incorrectly assigned atom types.

**Fig. 4:**
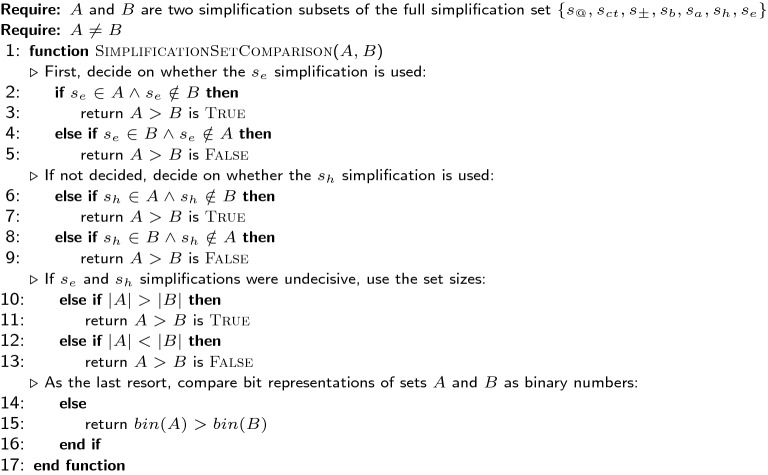
Comparison function used for sorting the list of simplification sets to determine the order of their application

Combinations inside each category are ordered according to the number of simplifications. This way combinations with fewer simplifications are tested before those having more. When ordering combinations of the same category with the same number of simplifications, their values of the *bin*() function are sorted as binary numbers in ascending order. The pseudocode of this algorithm is given in Fig. 4.

**Fig. 5:**
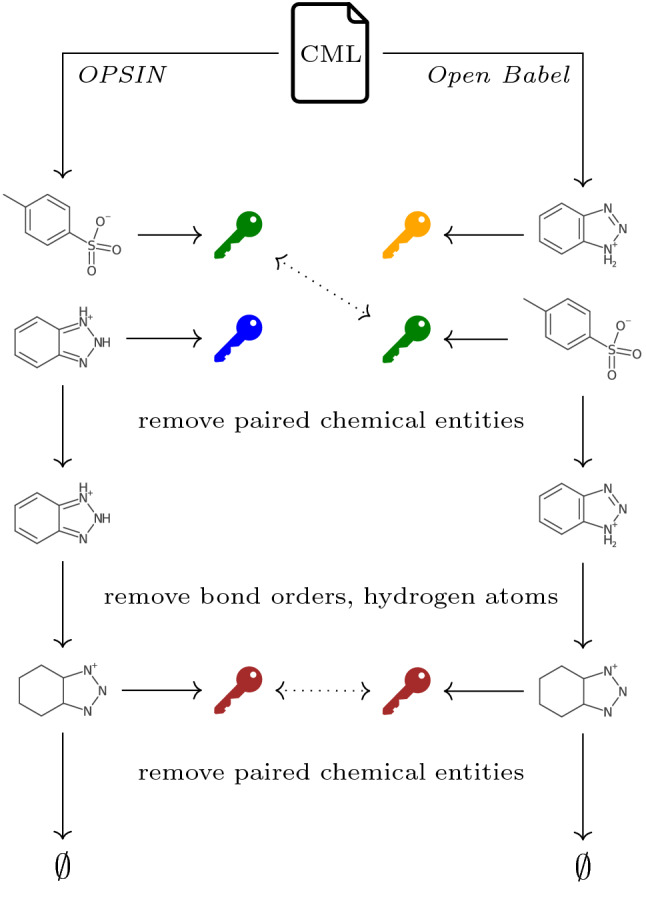
Cross-checking of the two chemical descriptions extracted from the same CML file corresponding to the COD entry 2239455 (differences discussed in Section S1.7 of Additional file 1). Two molecular entities are identified both from the chemical name (by *OPSIN*) and from the coordinates (by *Open Babel*). Canonical representations (“keys”) are then generated for unmodified molecular graphs, with identical representations marked with the same color in the figure. Molecular entities with matching keys are then removed and combinations of simplifications are applied until the keys of remaining molecular entities become identical (unsuccessful combinations of simplifications are not shown here for brevity)

The first of the combinations leading to identical representations in the tested sets of molecular entities is then recorded as minimal and the testing procedure is terminated. An illustration of cross-checking procedure is given in Fig. 5. It may happen that after applying some combination of simplifications two or more molecular entities in the same set become identical. For example, stripping chiral markers from enantiomers in a racemic mixture would render them identical molecular entities with an undefined chirality. In such case identical molecular entities are collated together, leaving again only the distinct molecular entities in the set. If at some point one set of molecular entities becomes a strict subset of the other, it is judged that the larger set has superfluous molecular entities and the matching procedure terminates. This allows us to identify possibly superfluous or missing molecular entities in the compared sets.

More simplifications similar to atom type removal could be introduced to ease the detection of common problems, for example, different attachment positions of methyl groups in rings. However, inclusion of such tests comes with the price of increased computation time.

### Canonical representation in SMILES

Several canonicalisation methods for SMILES have already been proposed [17, 19]. We have based our method on the canonical numbering established using *nauty* and implemented a stand-alone canonicaliser as the smi_canonicalise script in the *smiles-scripts* 0.2.0 software package.

To generate SMILES, a depth-first traversal is initiated through the chemical graph starting from the first atom of the established order. The same order is consulted each time the traversal has to pick the next atom from a set of candidates. Subsequently, all atom and bond attributes are added to the constructed representation. Cis/trans markers are added in a way that the first cis/trans marker in SMILES would be “/”. That is, if in the final SMILES representation the first cis/trans marker is “\”, all markers are flipped. Chiral markers are added taking into account the new order of atoms in the representation.

SMILES representations written by *Open Babel* [22] and Dassault Systèmes’s (formerly Accelrys) *Pipeline Pilot* [23] sometimes contain tetrahedral chiral centres with only three attachments to represent the participation of lone pairs of electrons in such centres, playing the role of an extra attachment to the central atom. None of the SMILES specifications mention lone pairs and the manually curated set of COD SMILES does not include such tetrahedral chiral centres. However, they are visible in the ChEMBL SMILES collection. We have prepared smi_canonicalise to read and write SMILES with lone pairs in tetrahedral chiral centres. If such chiral atom starts a SMILES representation, it is understood that the lone pair is the first attachment in the given (clockwise or counter-clockwise) enumeration. Otherwise, the lone pair is understood as the second attachment in the enumeration [24].

Aromaticity depiction is also an issue when attempting to achieve canonical representation in SMILES as there are two alternative ways of describing aromaticity, the aromatic and the so-called Kekulé representation. The aromatic representation marks up islands of delocalised bonds in a molecular entity with special attributes on atoms or bonds (or both), whereas Kekulé representation assigns alternating single/double bonds in these islands. For some molecular entities several alternative formally nonequivalent valid Kekulé representations can be produced due to possible alternative placement of single/double bonds. OpenSMILES specification allows using both Kekulé and aromatic representations, although the aromatic representation is preferred for output. Nevertheless, some cheminformatics programs, such as *OPSIN*, write SMILES using the Kekulé representation. In principle aromatic and Kekulé representations are interconvertible and to achieve the canonical representation a single representation should ideally be output. It might seem easier to convert an aromatic representation to its counterpart than the other way around since the locations of aromatic fragments in the molecular entity are explicitly provided. However, distributing single and double bonds in such fragments is not straightforward, moreover, possible alternative placement hinders canonical representation [25, 26]. Conversion of Kekulé form to aromatic is more desirable for canonicalisation due to the absence of alternative representations. There have been several attempts to devise algorithms for canonical representation in Kekulé form, for example, Richard L. Apodaca’s electron cycle detection algorithm [26] which requires finding all cycles in a graph. However, implementation and application of such algorithms are out of scope for the current study. In order to reduce the impact of the convention for aromaticity depiction, we have implemented an optional conservative kekulisation filter in smi_canonicalise. The filter is turned on by the --kekulise command line option which is off by default. Operating on the aromatic SMILES form, the filter locates non-fused even-numbered rings of aromatic atoms and represents them with alternating single and double bonds. When applied to the manually curated SMILES prior to comparisons, the conservative kekulisation filter yielded much better agreement between representations (see “Pairwise comparisons”).

It should be noted that our intention is not to propose a yet another method of writing canonical SMILES. To establish canonical atom numbering, a mathematically unambiguous algorithm would be preferred, but developing such algorithm is outside the scope of this study. In our study we use *nauty* which gives immutable canonical atom numbering with a fixed version of the *nauty* package, allowing us to compare chemical representations using a mathematically well-defined procedure of graph canonical representations.

### Chemical perception of crystallographic data

Crystallographic data provided in CIF format normally does not contain an explicit description of the chemical structure. A common way to augment crystallographic data with chemical attributes is to infer them from the atomic coordinates using heuristic-based methods. Chemical perception programs such as *Open Babel* often rely on this approach to assign chemical bonds, aromaticity, functional groups and so on. In this work, we use a chemical perception program called cif-perceive-chemistry [27, 28] which utilises the *OpenChemLib* cheminformatics library [29, 30] and was developed in-house to provide greater control over the specifics of the applied algorithm. The algorithm of cif-perceive-chemistry is similar to the Roger Sayle’s method [31] for small-molecule ligand extraction from PDB files, however, it aims to address the much greater chemical variety observed in the entirety of small-molecule crystal structures. The cif-perceive-chemistry program is highly specialised and currently only accepts stoichiometrically correct crystal descriptions in CIF format as input, generated by the cif_molecule program from the *cod-tools* software package [32]. The output of cif-perceive-chemistry is in SDF format.

### Converting SDF to SMILES

Representing chemically annotated 3D structures (for example, in CIF or SDF format) as SMILES is a complex task. While an expert chemist performs this task rather efficiently, there are several steps that are not that simple to automate and can only be performed by an expert. Furthermore, certain aspects of SMILES remain underspecified. In 2016 an open specification describing SMILES, called OpenSMILES, was released, clarifying the initial specification [33]. There are ongoing efforts to further the process of this clarification, namely SMILES+ [34] and *Dialect* [35], but none of them are complete at the moment. In this study, we are using OpenSMILES specification of SMILES. We use SMILES to produce a canonical representation of molecular entities (explained in detail in “Canonical representation in SMILES”). To perform a faithful conversion of molecular entity representations from SDF to SMILES, we have developed the sdf-to-smi tool which is distributed as part of the *smiles-scripts* software package [11, 36].

One of the issues addressed by sdf-to-smi is the identification of tetrahedral chiral centres. Initially all atoms with four *distinct* neighbours are treated as potential tetrahedral chiral centres. However, spatial arrangement has to be considered too as atoms in planar or near-planar arrangements should not be marked as chiral. Therefore, to evaluate if an atom with four neighbours should be treated as chiral, we measure the chiral volume of its normalised vectors. If the resulting volume is greater than 0.25 Å$^3$, we treat them as chiral and ignore otherwise. We have derived the threshold of 0.25 Å$^3$ by analysing chiral volumes of palladium and platinum atoms with the coordination number of four in the COD since such complexes are almost invariably square-planar.

Another important issue is that the SMILES format has no provisions to depict metal coordination bonds. Therefore, such bonds are either shown as single covalent bonds or not shown as bonds at all. There had been calls to introduce a special type of bond, the so-called zero-order bond [37], into chemical representation formats, but they have not been implemented in SMILES so far [38]. The sdf-to-smi program addresses this SDF conversion ambiguity by initially replacing all metal coordination bonds with single covalent bonds and then assigning higher bond orders based on the formal charges of the bonded atoms. If both atoms have non-zero formal charges of opposite signs, the bond order is adjusted to match the lowest of their absolute values. After that, the formal charges of the bonded atoms are reduced by the magnitude of the bond order. As a result, some of the bonded atoms may end up with a residual formal charge, however, the sign of the charge is never switched.

### Interpretation of chemical names

Accompanying chemical names can also be used to cross-check chemical descriptions of other types, as standardised chemical names have the ability to carry unambiguous machine-readable descriptions of chemical substances. For example, CIF files usually contain chemical names in IUPAC nomenclature, which could be used to check chemical perception results. IUPAC nomenclature is widely used in crystallographic reports, and software tools like *OPSIN* have been demonstrated to understand it with high level of success [11]. There are tools as well to identify chemical names in publication titles or full texts [39], but we did not use them in this study. Prior to parsing author-provided chemical names from CIF data items (_chemical_name_systematic and _publ_section_title) or CML <molecule> element name attributes with *OPSIN* 2.4.0, we subject them to automated regularisations making the names easier to read for *OPSIN*. First of all, symbols such as string-surrounding quotes and terminating dots are stripped. Then suffixes defining mixture proportions (both numeric and as the word “solvate”) are removed since *OPSIN* does not seem to correctly parse all of them. After that an attempt is made to convert CIF superscript notation (“ $\hat{}$  ...  $\hat{}\,\,$”) into the one understood by *OPSIN*. Finally, corrections of widespread minor spelling mistakes are done, such as replacing “napthalen” with “naphthalen”. Nearly 2500 of 128,600 chemical names in the COD revision 255755 CIF files were found to contain unbalanced parentheses. These occurrences are clearly errors, albeit needing expert curation to be fixed. In total, around 46,000 chemical names from COD CIF files and 2700 from CML files were successfully converted to SMILES representations by *OPSIN* and were used to cross-check other chemical descriptions.

## Results and discussion

### Pairwise comparisons

We have compared chemical annotations from several sources using the methods described in “Methods”. The compared pairs are indicated with bidirectional arrows in Fig. 1. An overview of numbers of entries participating in each of the comparisons is provided in Table 1. A table listing comparison results for each COD entry is provided in Additional file 2. Detailed analyses of the performed pairwise comparisons are described in Section S1 of Additional file 1.

Out of over 195,000 COD entries included in at least one comparison, almost 31,500 were detected as outright isomorphic in every comparison they participated. Over 144,500 entries needed simplifications to be detected as isomorphic in at least one comparison, but in every comparison they were included they eventually reached isomorphism.

As expected, difference in aromaticity representation caused many pairs of SMILES to be judged as mismatching. Pairs differing in representation form (aromatic versus Kekulé) are usually rendered isomorphic after removal of aromaticity settings and converting all bond orders to single. Pairs differing in Kekulé representations require converting all bond orders to single to arrive to isomorphic forms. To minimise the impact of the convention for aromaticity depiction, we have employed a conservative kekulisation filter (described in “Canonical representation in SMILES”) in two of the comparisons against the manually curated SMILES set as it uses the aromatic form. The filter is applied to the manually curated SMILES prior to the comparison. This simplistic method greatly reduced pairs of SMILES differing in aromaticity and bond order attributes in both comparisons.

The ambiguity of the representation of metal coordination bonds in SMILES greatly influenced the number of mismatches in the comparison of manually curated SMILES and coordinate-derived annotations that could not be explained automatically. As a result, nearly 10% of all participating entries have such mismatches. In most cases manually curated SMILES have greater connectivity than the coordinate-derived annotations.

Differences in chirality mostly arose due to chemical names not being specific enough in regard to the stereochemistry. In addition to that, some of the annotations derived from crystallographic data describe only one specific stereoisomer instead of a racemate due to the crystal belonging to a non-Sohncke space group [40]. This issue can be resolved by checking the space group.

Our approach also helped to identify more serious issues in chemical annotations that did not arise from the differences in representation (see Sections S1.2–S1.7 of [Additional file 1] for per-case analyses). To test it, we have manually analysed over 30 mismatches and confirmed that around 20 of them are in need of curation (see Sections S1.2–S1.7 of Additional file 1 for per-case results). In most cases, pairs that had mismatching atom types or that could not be automatically simplified to an isomorphic representation highlighted obviously incorrect chemical structure assignments. For example, analysis of COD entry 1549674 [41] allowed to identify contradictions not only in the different chemical representations associated with this entry, but also in the text of the original peer-reviewed publication. The entry in question describes a molecular entity derived from *spiroisoxazoline*, however, different representations of this entity give different attachment locations for a nitro group. The publication text, 3D depiction of the structure in the publication and the crystal structure in the CIF all describe its parent as *4-nitrophenyl* while the publication title, 2D depiction of the structure in the publication, chemical names given in the publication text, CIF and CML files as well as the chemical structure depicted in the CML file describe it as *2-nitrophenyl*. We have reported this mismatch to the Editorial Office of *IUCrData* and received an acknowledgement that a corrigendum will be published. We suggest that the inclusion of similar automated cross-checks into the publication process would help to more easily identify discrepancies during the review stages.

The lack of unambiguous machine-readable identifiers of compounds in publications (as discussed in “Data sources”) hinders comparisons since it requires assumptions in order to reconstruct the missing mapping. It also makes it more difficult for reviewers to double-check the correctness of chemical names and structures in the data files, explaining the observed discrepancies between statements in the CML data and the text of the main publication. It is suggested that journals adopt publication rules mandating machine-readable mappings such as IUPAC FAIRSpec [42]. Such policy would make reviewing supplementary data easier and eliminate certain mistakes from the publication more readily.

### Comparison of curated and chemical name-derived SMILES

In a previous work, we have attempted to compare curated [11] and *OPSIN*-derived SMILES representations using *OPSIN* 2.3.0 to convert chemical names to SMILES and *Open Babel* 2.2.3 to perform their standardisation prior to comparison. Out of over 30,000 pairs of representations, 1167 were found to be different with the reason for such difference not being found in an automatic way. With the new methodology, we repeated the comparison for over 34,500 pairs of representations to find approximately twice as many discrepancies that could not be explained automatically. This increase is mostly attributable to the stricter matching imposed by the novel algorithm: no attempts are made to detect missing atoms or resolve differences in molecular entity counts. In two cases, pairs of representations were reclassified from identical to differing due to reasons that could not be identified automatically. In COD entry 2011092, oxygen attachment site is incorrectly located by *OPSIN* 2.4.0 (it is located correctly by *OPSIN* 2.3.0). Mismatch in COD entry 2203592 is a side effect of pre-processing chemical names before passing them to *OPSIN* (see “Interpretation of chemical names”). The influence of both the *OPSIN* version change and pre-processing chemical names was therefore found to be negligible. The increase of total number of pairs from 30,000 to over 34,500 is due to the growth of both the COD and the manually curated set of SMILES representations. The summary table of the comparison is shown in Table S1 of Additional file 1.

### Chemical perception of the COD data

We have performed chemical perception with the workflow described in  “Chemical perception of crystallographic data” for all entries of the COD in revision 247260 (around 450,000 structures). Chemical annotations were derived for around 68% of entries. The remaining crystal structures were not processed for the following reasons:

- 22% of all entries were identified as describing polymeric structures. Here by polymer we mean molecular entities spanning an infinite number of crystal unit cells. While polymers are easily expressed in CIF format, the SMILES format lacks the means to represent periodicity. Moreover, there are no guidelines for selection of representational units for polymeric molecular entities. Due to these reasons we decided to skip polymeric structures.
- 8% of all entries contained either steric clashes (bumps) or unreasonably high valencies. Such violations usually mean unmarked structural disorder or incorrect definition of crystal symmetry.
- 2% of all entries either contained no atomic coordinates or contained atomic coordinates that were too complex to be processed due to the limitations of the employed workflow. These limitations include restrictions on the computational resources such as CPU time and RAM as well as data format limitations, i.e. the MDL Molfile V2000 format is not suited to describe molecular entities with more than 999 atoms or 999 bonds in a single file.

All generated chemical annotations were subsequently validated by the same cif-perceive-chemistry program against a set of molecular data consistency tests. Some of the tests relied on hard-coded rules such as “crystals should have an overall neutral formal charge” or “carbon atoms should not have valencies higher than 4”. Other tests were based on bond length statistics derived from previously observed valid 3D chemical structures, for example, “the length of a double bond between sulphur and oxygen atoms should be in range [*x*, *y*]”. 25% of all annotations violated at least one of these tests, either due to errors in the original CIF (e.g. unmarked atom disorder) or due to the current limitations of the cif-perceive-chemistry program. However, it should be noted that the validation rules were designed to err on the side of caution thus some legitimate non-classically bonded structures such as carboranes may have been incorrectly reported as invalid. In this work all of the generated chemical annotations were used in comparisons regardless of the molecular data consistency test results.

### Testing the proposed method with ChEMBL data

To test the proposed method for SMILES canonicalisation, we performed a duplicate search in the ChEMBL database. ChEMBL is a database of distinct chemical compounds, thus it should not contain duplicates. Finding duplicates in it would highlight problems with our methods or, with a much lower probability, genuine identical entries in ChEMBL. A similar study has been carried out by Noel O’Boyle, the developer of the Inchified SMILES and Universal SMILES [17].

We have subjected all SMILES representations taken from ChEMBL versions 13 (released on 2012-02-29) and 29 (released on 2021-07-01) to canonicalisation using methods described in this paper. Version 13 has been chosen to compare the findings with those reported by O’Boyle and version 29 was the latest release at the time of the test. Both versions of ChEMBL database have entries with identical original SMILES (attached as Additional files 3 and 4). This is most likely due to SMILES not being the primary storage format for ChEMBL data, thus some chemical attributes are lost during conversion into SMILES on ChEMBL side. Inspection of some groups of ChEMBL entries with identical original SMILES revealed that corresponding InChI representations are different at stereochemical layers. Since identical input SMILES will result in identical canonicalised ones, we treat them as false-positive duplicates and exclude from further analysis.

**Fig. 6:**
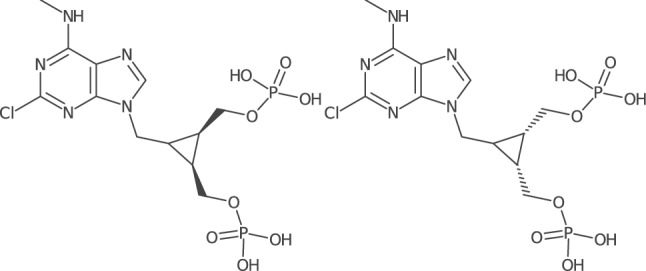
Depiction of SMILES representations for ChEMBL version 13 entries CHEMBL106860 (left) and CHEMBL323265 (right). Structural formulas are drawn with cdkdepict from *smiles-scripts* using *CDK* [44]

Three pairs of duplicates were found in ChEMBL version 13 (attached as Additional file 5). The first of them, CHEMBL106860 and CHEMBL323265, differs in the settings of two chiral centres on cyclopropane ring (Fig. 6). Due to the setting of the third chiral centre on cyclopropane ring being undefined, it is impossible to distinguish these two ChEMBL entries from each other in SMILES representations. SDF files for these entries each have three marked atom stereo centres and three stereo bonds, thus their indistinguishability is most likely limited to SMILES. Both entries CHEMBL106860 and CHEMBL323265 are retained in ChEMBL version 29, but the chiral setting is added for cyclopropane carbon linked to the purine moiety in SMILES of both entries. This addition thus distinguishes these two molecules from each other in SMILES representations. Atom stereo centres have disappeared from SDF files of ChEMBL version 29, but the same values of stereo bonds have been retained. Each of the remaining two pairs of duplicates, one being CHEMBL1213498 and CHEMBL1213499, and the other CHEMBL1213545 and CHEMBL1213546, represents a pair of diastereomers of a tetraoxane derivative. Such derivatives contain three carbon atoms linked to two identical branches, but with a configuration around them that is relevant to establish the actual isomer, in a similar way to the carbon atom linked to the heterocycle in the cyclopropane ring in the previous pair of CHEMBL106860 and CHEMBL323265. The two compounds of each pair are different according to their SDF representations and the original publication [43], but their SMILES representations in ChEMBL version 13 differ in the configuration of two of the three carbon atoms, thus rendering the two SMILES representations equivalent (they should differ in the configuration of just one carbon atom to faithfully represent the two diastereomers), opposed to the corresponding SDF representations which differ in the setting of just a single stereo bond as expected. Hence we conclude that the SDF representations are correct, but one of the SMILES representations is wrong, triggering the occurrence of a false duplicate. Comparing to ChEMBL version 29, we find the SDF representations identical to those in version 13, whereas SMILES representations each have lost the configuration setting in one of the three relevant chiral carbon atoms, rendering these SMILES incomplete, which triggers the occurrence of false duplicates too.

Nine pairs of duplicates were found in ChEMBL version 29 (attached as Additional file 6). Of these nine, eight concern molecules very similar to the aforementioned molecules with tetraoxane rings. However, as explained in the previous paragraph, in these cases the tetraoxane carbon atom belonging to ethyl- or methylcyclohexane moiety has lost its chirality setting in SMILES representations. As a result, our method does not get enough information to consider these molecules as distinct. The remaining pair of duplicates, CHEMBL1512909 and CHEMBL2068737, both describe molecules containing an 8-membered ring, but differ in a cis/trans setting in this ring. Entry CHEMBL1512909 has cis/trans setting for a single double bond in this ring while CHEMBL2068737 does not have any cis/trans settings. Our method does not consider cis/trans settings in rings of size 8 or less (same as in O’Boyle’s application [17]), therefore these two molecules are considered identical.

O’Boyle found six duplicates in ChEMBL version 13 using Inchified SMILES and 18 more using Universal SMILES, while we found six duplicates (three pairs) using our own method. Their publication explicitly lists only two pairs of duplicates, one being CHEMBL1180158 and CHEMBL186139, and the other CHEMBL1512517 and CHEMBL1730955, both pairs having identical original SMILES and thus excluded from our analysis. It is important to note that O’Boyle used SDF files as the input to produce canonical SMILES representations for the test while we attempted canonicalisation of SMILES provided by ChEMBL. Thus, our test detects only entries which have isomorphic structures as encoded in readily available SMILES representations. From these findings we conclude that our method for duplicate detection yields negligible amount of false positives and thus is suitable for the investigation of chemical annotations in the COD. In principle the test could also be extended with SDF files, either by converting them to SMILES using sdf-to-smi (a possibly lossy approach) or by constructing molecular graphs directly from the SDF files.

## Conclusions

In this publication, we present an application of graph isomorphism algorithms on chemical graphs, with a novel approach to the representation of stereochemistry using additional graph vertices and edges. It was shown that overlaying multiple chemical graphs allows to compare their corresponding chemical attributes and that mismatches in such comparisons can help detect both the differences in the used notations as well as the more legitimate data discrepancies. Gradual simplifications of chemical graphs facilitate the identification of the minimal set of differences in the aforementioned cases.

Overlay and comparison of molecule descriptions collected from peer-reviewed publications provides insight into the usability of such data for chemical data analysis. It is clear that conflicting notation conventions render chemical representations difficult to compare. Various convention converters may be employed to produce canonical representations, however, they usually involve heuristics which are not free from overinterpretations. Mistakes in both the crystallographic and the chemical representations may hinder the usability even more. Nevertheless, mismatches of overlaid molecule representations could be highlighted as “unusual” and passed for data curators. To illustrate this approach, we have manually analysed over 30 mismatches and have concluded that around 20 of them are in need of curation. We have reported one of these structures to its publisher who promised to publish an errata. Application of the described methods prior to publication could improve the consistency of both the published chemical annotations and publication texts.

With this publication we provide a compendium of pairwise comparisons of different representations of data from the COD. This compendium could be consulted to select entries with high level of agreement between their representations. Alternatively, entries with mismatches are of interest for purposes of both data curation and development of the comparison algorithm itself.

**Table 1:** Overview of numbers of pairs participating in each comparison

Comparison	Isomorphic without simplifications	Isomorphic with simplifications	Not isomorphic	Total
Curated SMILES / Chem. name from CIF	9754	22,720	2196	34,670
Curated SMILES / Coordinate-derived annot.	31,757	140,436	15,944	188,137
Chem. name from CIF / Coordinate-derived annot.	15,630	20,807	3199	39,636
Chem. name from CIF / Chem. name from CML	1453	66	14	1533
Chem. name from CIF / Chem. name from title	22,022	337	190	22,549
Coordinate-derived annot. / Chem. name from CML	592	904	55	1551
Chem. name from CML / CML-encoded annot.	1817	19	13	1849

“Isomorphic without simplifications” gives the number of pairs considered outright isomorphic, “Isomorphic with simplifications”–convertible to isomorphic form by a series of simplifications and “Not isomorphic”–not convertible to isomorphic. Detailed breakdowns of used simplifications are given in Tables S1–S7 of Additional file 1

## Supplementary Information

**Additional file 1. **Overview of comparison results.

**Additional file 2. **Comparison results for COD entries.

**Additional file 3. **ChEMBL version 13 entries with identical SMILES.

**Additional file 4. **ChEMBL version 29 entries with identical SMILES.

**Additional file 5. **ChEMBL version 13 entries perceived as identical.

**Additional file 6. **ChEMBL version 29 entries perceived as identical.

## Data Availability

The datasets supporting the conclusions of this article are available in the Crystallography Open Database, https://www.crystallography.net/cod/. Curated SMILES are available at https://www.crystallography.net/cod/smi. All data in the COD is dedicated to the public domain and licensed under the CC0 License. Analysed CML files are accessible at publishers’ websites. *smiles-scripts* is released under GNU GPLv2 license and can be obtained free-of-charge from svn://www.crystallography.net/smiles-scripts. This paper refers to version 0.2.0 of *smiles-scripts*, source of which is available at https://www.crystallography.net/cod/archives/2022/software/smiles-scripts/smiles-scripts-0.2.0.tar.gz.
