# Supplementary material for: Graph isomorphism-based algorithm for cross-checking chemical and crystallographic descriptions
Source: J Cheminform. 2023 Feb 23;15:25. doi: 10.1186/s13321-023-00692-1 (PMC9948373; doi:10.1186/s13321-023-00692-1)
Supplement: Supplementary file 1 — Additional file 1. Overview of comparison results. [file 13321_2023_692_MOESM1_ESM.pdf]

Graph isomorphism-based algorithm for cross-checking chemical and crystallographic descriptions

Supplementary material

Andrius Merkys[1*], Antanas Vaitkus[1], Algirdas Grybauskas[1], Aleksandras Konovalovas[2], Miguel Quirós[3], and Saulius Gražulis[1]

[1]Sector of Crystallography and Chemical Informatics, Institute of Biotechnology, Life Sciences Center, Vilnius University, Saulėtekio al. 7, LT-10257, Vilnius, Lithuania
[2]Department of Biochemistry and Molecular Biology, Institute of Biosciences, Life Sciences Center, Vilnius University, Saulėtekio al. 7, LT-10257, Vilnius, Lithuania
[3]Departamento de Química Inorgánica, Universidad de Granada, 18071, Granada, Spain
[*]*E-mail: andrius.merkys@gmc.vu.lt*

December 29, 2022

S1 Comparisons

S1.1 Curated SMILES and Chemical names from CIF

As described in Section 4.2 of the main text, we have compared curated and *OPSIN*-derived SMILES representations from CIF files. The results of this comparison are analysed in Section 4.2. The summary table of the comparison is shown in Table S1.

S1.2 Chemical names from CIF and publication title

To assess the quality of substance chemical names in publication titles and CIF files themselves, we have employed *OPSIN* tool to parse chemical names in publication titles (`_publ_section_title` CIF data item) and provided chemical names (`_chemical_name_systematic` CIF data item). Prior to processing with *OPSIN*, publication titles were processed to remove words that usually precede

#	H atoms	aromaticity	atom types	bond order	charge	chirality	cis/trans	superfluous mol.
9754	isomorphic							
8807		×		×				
4490				×				
2344				×	×			
1436						×		
1019		×		×	×			
1000							×	
544	×				×			
396				×		×		
376		×		×		×		
348	×			×				
297								×
236	×			×	×			
176	×	×		×	×			
158	×	×		×				
152				×	×	×		
120		×		×				×
74	×		×	×				
73				×				×
68					×			
57						×	×	
51	×							
42			×	×				
39		×		×	×	×		
38				×	×			×
31			×					
28	×		×	×	×			
26	×		×					
26			×		×			×
23	×				×			×
2196	cannot be explained automatically							
245	not shown here for brevity							
34670	total							

Table S1: Comparison of curated SMILES and SMILES derived from chemical names from CIF files. Each row pertains to a class of pairs differing in a set of chemical attributes, marked with "×". Column "#" lists the numbers of such pairs. The underlined value corresponds to pairs considered identical. Row "cannot be explained automatically" lists pairs for which the reason of the mismatch could not be recognised automatically.

or follow chemical names. Afterwards, we have compared SMILES representations derived from the publication titles with those derived from chemical names given in CIF files. Summary of the comparison results are provided in Table S2.

As expected, most of the pairs were isomorphic. Nevertheless, it is interesting to observe some differences, mostly arising due to publication titles providing substance names with different level of detail. We have manually investigated a few mismatching cases:

- COD entries 2212904 and 2207621 were correctly detected as differing only in chirality. For the former, title and chemical name provide opposite chiralities. The title of the latter one gives *(R*)-Methyl 3-carboxy-2-hydroxypropanoate* while chemical name in CIF file is *Methyl 3-carboxy-2-hydroxypropanoate*.
- COD entry 2233090 is correctly categorised as differing in H atoms and atom types. Its chemical name from CIF file incorrectly describes the five-membered ring as cyclopentane, while the ring is actually imidazolidine, as seen both from the publication title and the coordinates.
- COD entries 2233352 and 2239411 are categorised as "cannot be explained automatically", which incidentally is the second most populous category in Table S2. For entry 2233352, the title does not account for second trimetylphenyl attachments for each boron atom in the structure. For entry 2239411, the chemical name in CIF file describes a completely different molecular entity.

S1.3 Chemical names from CIF and CML files

We have carried out the comparison of chemical structures extracted by *OPSIN* from chemical names provided in CML and CIF files. Due to the lack of author-provided mapping between CIF and CML files, which is described in Section 3.1 of the main text, we have combined the SMILES representations coming from CIF files of the same publication and compared them with combined SMILES representations coming from CML files of the same publication. Surprisingly, some of them differ hinting at apparently different quality control scrutiny levels applied to them. Summary of the comparison results are provided in Table S3.

Analysis of random entries revealed very different causes of mismatches:

- COD entry 1545041 is detected as isomorphic disregarding atom types due to the fact that a chlorine atom is not mentioned in the chemical name from the CIF file (replaced with an implicit hydrogen atom instead).
- The same category is assigned to COD entry 1558620 due to a thione designator incorrectly appearing in the chemical name from the CML file. This causes a substitution of a keto- group with a thio- group.
- CML file for COD entry 2241635 does not provide the setting for a tetrahedral chirality centre.

#	H atoms	atom types	bond order	charge	chirality	cis/trans	superfluous mol.
22022	isomorphic						
57	×			×			
50							×
48			×				
48					×		
38	×		×				
20						×	
12	×		×	×			
11		×					
11			×	×			
8	×	×					
6	×	×	×				
6	×	×		×			
3	×	×					×
3		×	×				
3				×			
2		×		×			
2		×		×			×
2					×		×
1	×						
1	×			×	×		
1	×			×			×
1	×		×		×		
1		×	×	×			
1		×				×	
1			×				×
190	cannot be explained automatically						
22549	total						

Table S2: Comparison of SMILES derived from chemical names from CIF files and publication titles. For the legend refer to Table S1.

#	H atoms	atom types	bond order	charge	chirality	cis/trans	superfluous mol.
1453	isomorphic						
25							×
9					×		
8	×		×				
6	×			×			
5						×	
4			×				
3		×					
1	×	×					
1	×			×			×
1		×	×				×
1		×					×
1					×	×	
1					×		×
14	cannot be explained automatically						
1533	total						

Table S3: Comparison of SMILES derived from chemical names from CIF and CML files. For the legend refer to Table S1.

- COD entry 1551060 is marked as isomorphic disregarding bond order due to different aromaticity representations of the same molecular entity in *OPSIN* output.
- Entries 2020968–2020972 originate from the same publication and are marked as isomorphic disregarding superfluous molecular entities as for the five single-molecular entity crystals only four CML files are provided in the supplementary material.

Analysed mismatches mostly highlight problems in chemical names arising in supplementary material. A much more detailed analysis could be performed if one-to-one mapping between CIF and CML files was available. We believe tighter connections between machine-readable representations (such as CIF and CML files) and crystals/compounds described in publications would greatly benefit the usability of these pieces of data.

S1.4 Curated SMILES and coordinate-derived chemical annotation

Summary of the comparison results are provided in Table S4. As almost 10% of entries have differences which cannot be explained automatically, we have analysed five entries from this category and five more from the others (i.e. ones resulting in eventual isomorphism).

The five analysed mismatches from the "cannot be explained automatically" category (COD entries 1549313, 2230878, 2239608, 4070104 and 7100277) are caused by different interpretation of metal coordination complexes. For them, manually curated SMILES have greater connectivity than the coordinate-derived annotations.

As in other comparisons, difference in aromaticity representation is detected here too. All five analysed mismatches in other categories were affected by this difference. Additional differences are as follows:

- The coordinate-derived annotation for COD entry 7200870 represents the azido group as having a triple bond and opposite formal charges on two of the nitrogen atoms while the manually curated SMILES represents it as having one double bond, one triple bond and no formal charge.
- For entry 1512357, the algorithm assigns chirality marker for the single tetrahedral chiral centre in the structure. However, since the entry is in *P-1* space group, both stereoisomers are present in the crystal. To handle such cases, the algorithm could be improved to detect non-Sohncke space groups and accordingly adjust the representation of chirality, as enantiomerically pure compounds can only occur in Sohncke space groups.
- Three entries, 1518599, 2237173 and 4110251, differ only in aromaticity depiction.

#	H atoms	aromaticity	atom types	bond order	charge	chirality	cis/trans	superfluous mol.
31757	isomorphic							
25295		$\times$		$\times$				
14124				$\times$				
11682				$\times$	$\times$			
9657	$\times$	$\times$		$\times$	$\times$			
6938		$\times$		$\times$	$\times$			
6579		$\times$		$\times$		$\times$		
5924	$\times$			$\times$	$\times$			
5806								$\times$
5491						$\times$		
5412	$\times$	$\times$		$\times$				
3792							$\times$	
3662	$\times$				$\times$			
3617		$\times$		$\times$	$\times$	$\times$		
2940	$\times$	$\times$		$\times$	$\times$	$\times$		
2838					$\times$			
2700	$\times$							
2584				$\times$		$\times$		
2336	$\times$			$\times$				
2323				$\times$	$\times$	$\times$		
2031				$\times$				$\times$
2004	$\times$			$\times$	$\times$	$\times$		
1711	$\times$	$\times$		$\times$		$\times$		
1420		$\times$		$\times$				$\times$
1334	$\times$				$\times$	$\times$		
1141	$\times$					$\times$		
1091	$\times$			$\times$		$\times$		
968				$\times$	$\times$			$\times$
657							$\times$	$\times$
575					$\times$	$\times$		
15944	cannot be explained automatically							
3804	not shown here for brevity							
188137	total							

Table S4: Comparison of curated SMILES with SMILES derived from atomic coordinates. For the legend refer to Table S1.

S1.5 Chemical name from CIF and coordinate-derived chemical annotation

We have also compared SMILES derived from chemical names with those inferred from coordinates. Summary of the comparison results are provided in Table S5. To evaluate the comparison results, we have examined five COD entries for which SMILES representations were not immediately isomorphic. Three of them, COD entries 2233542, 2234827 and 2211700, had cis/trans settings derived by coordinate-based approach, albeit missing in their chemical names. Other differences:

- The difference for entry 2105650 is caused by SMILES representations corresponding to different charge distributions in the molecular entity: `cif-perceive-chemistry` produces a zwitterionic form, whereas *OPSIN* derives the neutral form from the chemical name.
- Entry 2211700 has the same issue with a single algorithmically detected chiral centre in *P-1* space group as entry 1512357 described in Section S1.4.
- Entry 2234827 had different kekulisation forms assigned by *OPSIN* and `cif-perceive-chemistry`.
- The crystal structure of entry 2242110 contains extra hydrogen atoms which contribute to incorrect overall charge assignment.

S1.6 Chemical name from CML and coordinate-derived chemical annotation

To cross-check chemical annotations from CML files and coordinate-derived ones, we have used *OPSIN* to convert chemical names from CML to SMILES and compared them with those originated from coordinate-derived annotations using `sdf-to-smi`. Summary of the comparison results are provided in Table S6.

To analyse the mismatches, we have taken three pairs marked as "cannot be explained automatically" and three pairs marked as having "superfluous molecular entities". Mismatches classified as "cannot be explained automatically" mostly happened due to problematic chemical names in the CML files:

- One carbon atom is missing in each of CML files of COD entries 2020720–2020722, containing *4,5,6,7-tetrahydro-1H-pyrazolo[4,3-c]pyridines*. Carbon atoms are missing from both the chemical names and the structures in the aforementioned CML files.
- For 1549674, the publication describes a molecular entity derived from *spiroisoxazoline*, however, different representations of this entity give different attachment locations for a nitro group. The publication text, 3D depiction of the structure in the publication and the crystal structure in the CIF all describe its parent as *4-nitrophenyl* while the publication title, 2D depiction of the structure in the publication, chemical names given in

#	H atoms	atom types	bond order	charge	chirality	cis/trans	superfluous mol.
15630	isomorphic						
7574			×				
3521					×		
2624						×	
1360			×		×		
1198			×	×			
914							×
888	×			×			
460	×		×	×			
348					×	×	
336	×		×				
164			×	×	×		
162					×		×
143			×				×
112						×	×
78	×			×			×
76	×						
69	×			×	×		
68	×	×		×			×
61	×	×	×				
56	×	×					
52		×		×			×
48				×			
42			×	×			×
40	×		×	×			×
40	×		×		×		
38		×					
31		×	×				
27	×		×	×	×		
26	×	×	×	×			
3199	cannot be explained automatically						
251	not shown here for brevity						
39636	total						

Table S5: Comparison of SMILES derived from chemical name from CIF and coordinate-derived chemical annotations. For the legend refer to Table S1.

the publication text, CIF and CML files as well as the chemical structure depicted in the CML file describe it as *2-nitrophenyl*. We have reported this mismatch to the Editorial Office of *IUCrData* and received an acknowledgement that a corrigendum will be published.

- In 2238972, chemical name of a chloroform molecular entity is correctly interpreted in the CML file. However, in the crystal structure chloroform is placed too close to an inversion centre, causing incorrect connectivity with its own symmetry equivalent. Such cases are not uncommon in the COD and ideally should be resolved by declaring such molecular entities as disordered around a special position.

Two of the groups marked as having "superfluous molecular entities" indeed lack constituents in chemical names provided in their CML files:

- The group consisting of COD entries 2241571–2241573 misses a *1-aza--8-azoniabicyclo[5.4.0]undec-7-ene 2-hydroxy-3,5-dinitrobenzoate* molecular entity.
- CML file for entry 2021432 does not contain the acetonitrile molecular entity present in the crystal structure. Acetonitrile is widely used as a solvent and solvent molecular entities are rarely of interest both for crystallographers and for chemists. Thus, it is not an unusual practice to exclude them from descriptions of crystal structure and contents.

A more problematic situation is observed in COD entry 1542900. The asymmetric unit of the crystal contains two chemically identical molecular entities. Therefore, their chemical names are the same and the same name is also used in the CML. However, upon chemistry perception from the crystal these two molecular entities are assigned different Kekulé representations, hence not immediately understood as identical by our method. The method recognises one of these representations as the one mentioned in the chemical name provided in the CML, but the remaining molecular entity is left unpaired. Thus, this entry is identified as having "superfluous molecular entities".

S1.7 Chemical name from CML and CML-encoded annotation

It is interesting to compare how chemical names match chemical structures in CML files provided in the supplementary material of peer-reviewed publications. The summary table for this comparison is shown in Table S7. We have manually inspected ten groups not outright considered isomorphic:

- Differences in kekulisation forms were detected in two groups, namely, COD entries 2240071 and 2241457. The rest of the groups display more serious mismatches between the described molecular entities.

#	H atoms	atom types	bond order	charge	chirality	cis/trans	superfluous mol.
592	isomorphic						
381			×				
126					×		
88						×	
53							×
51			×	×			
47			×		×		
26	×			×			
23	×		×				
17					×	×	
14	×		×	×			
14			×				×
12						×	×
10					×		×
4			×		×		×
3	×	×					
3	×	×		×			×
3	×			×			×
3		×	×				
3			×	×	×		
2	×	×	×				
2	×		×	×			×
2		×			×		×
2			×	×			×
2					×	×	×
1	×						
1	×	×	×	×			
1	×	×					×
1	×		×				×
1	×			×	×		
55	cannot be explained automatically						
8	not shown here for brevity						
1551	total						

Table S6: Comparison of SMILES derived from chemical names from CML and coordinate-derived chemical annotations. For the legend refer to Table S1.

- For COD entry 1545011, CML file shows two OH attachments at S site. However, this is corroborated neither by its CIF file, nor by the figures in the original publication.
- CML file for entry 2020445 does not convey the same iodine–iodine connectivity as is relayed in its CIF file, systematic name and illustrations in the original publication. Instead, the CML file shows iodine atoms as unconnected. Moreover, the heterocyclic molecular entity present in the crystal is written three times in the CML file associated with this publication.
- CML file for 2239009 contains attached oxygen atoms while the crystal structure does not. The provided chemical name is correct.
- CML file for 2239455 has two hydrogen atoms attached to N1 of a triazole ring while in fact one of hydrogen atoms is attached to N2 instead.
- CML file for 2240048 shows different attachment locations of methoxyphenyl groups to the central terephthalate. Such structure is corroborated neither by CIF nor by figures of the original publication.
- The group of COD entries 2242193–2242195 contains the triaminoguanidine molecular entity, which is incorrectly represented in the CML file (incorrect connectivity). Furthermore, *OPSIN* also interprets triaminoguanidine as having different connectivity both from the true one as provided in the CIF file, and from the one present in the CML file.
- Group of COD entries 2242680–2242682 has been identified as isomorphic only when the H atoms, atom types and superfluous molecular entities are ignored. This is due to the incorrect connectivity perceived from the chemical name by *OPSIN*. Chemical structures in CML files of each entry in the group contain three molecular entities: asparagine, water molecule and a halogen atom which is different in each entry. *OPSIN* interprets asparagine molecular entities as having one of their carboxyl oxygen atoms replaced with halogens. This issue has been reported to the *OPSIN* developers and subsequently fixed.
- Chemical structures in CML files for COD entries 2243660–2243662 describe different molecular entities than those reported in chemical names and seen in crystal structures. The reason is an incorrect attachment site for acetamide group in benzoate-based molecular entities.

#	H atoms	atom types	bond order	charge	chirality	cis/trans	superfluous mol.
1817	isomorphic						
6	×			×			
3			×				
2	×	×					
2					×		
2						×	
1	×	×					×
1	×		×				
1		×					×
1							×
13	cannot be explained automatically						
1849	total						

Table S7: Comparison of SMILES derived from chemical names from CML and CML-encoded annotations. For the legend refer to Table S1.
